# Influence of TEGDMA monomer on MMP-2, MMP-8, and MMP-9 production and collagenase activity in pulp cells

**DOI:** 10.1007/s00784-020-03545-5

**Published:** 2020-08-26

**Authors:** Bálint Viktor Lovász, Edina Lempel, József Szalma, György Sétáló, Mónika Vecsernyés, Gergely Berta

**Affiliations:** 1 Department of Oral and Maxillofacial Surgery, University of Pécs Medical School, 5 Dischka Gy. St, Pécs, 7621 Hungary; 2 Department of Conservative Dentistry and Periodontology, University of Pécs Medical School, 5 Dischka Gy. St, Pécs, 7621 Hungary; 3 Department of Medical Biology and Central Electron Microscope Laboratory, University of Pécs Medical School, 12 Szigeti St, Pécs, 7624 Hungary

**Keywords:** Dental material, Composite resin, TEGDMA, Cytotoxicity, MMP, Total collagenase activity

## Abstract

**Objectives:**

Resin-based composites may leach monomers such as triethylene-glycol dimethacrylate (TEGDMA), which could contribute to intrapulpal inflammation. The aim of this investigation was to examine whether various concentrations of TEGDMA are able to influence dentally relevant Matrix metalloproteinase (MMP)-2, MMP-8, and MMP-9 production, total collagenase/gelatinase activity in pulp cells, and suggest possible signaling mechanisms.

**Materials and methods:**

Pulp cells were cultured, followed by a 1-day exposure to sublethal TEGDMA concentrations (0.1, 0.2, and 0.75 mM). Total MMP activity was measured by an EnzCheck total collagenase/gelatinase assay, while the production of specific MMPs and the relative changes of phosphorylated, i.e., activated signaling protein levels of extracellular signal-regulated kinase (ERK)1/2, p38, c-Jun N-terminal kinase (JNK) were identified by western blot. Immunocytochemistry image data was also plotted and analyzed to see whether TEGDMA could possibly alter MMP production.

**Results:**

An increase in activated MMP-2, MMP-8, and MMP-9 production as well as total collagenase activity was seen after a 24-h exposure to the abovementioned TEGDMA concentrations. Increase was most substantial at 0.1 (*P* = 0.002) and 0.2 mM (*P* = 0.0381). Concurrent p-ERK, p-p38, and p-JNK elevations were also detected.

**Conclusions:**

Results suggest that monomers such as TEGDMA, leached from resin-based restorative materials, activate and induce the production of dentally relevant MMPs in pulp cells. Activation of ERK1/2, p38, or JNK and MMP increase may play a role in and/or can be part of a broader stress response.

Clinical relevance

Induction of MMP production and activity may further be components in the mechanisms of intrapulpal monomer toxicity.

## Introduction

Matrix metalloproteinases (MMPs) are zinc- and calcium-dependent protein hydrolases belonging to the “metzincin” group of metallopeptidases [1]. Various types of MMPs have distinct but overlapping specificities, in particular collagen/laminin-specific collagenases (e.g., MMP-1, MMP-8) and gelatinases (e.g., MMP-2, MMP-9) seem to play the biggest role in dental pathology [2, 3]. Such MMPs have been known to be produced by pulp cells and found in sound dentine [4], suggesting a physiologic function. Gelatinases MMP-2 and MMP-9 are believed to have a wide range of extracellular matrix (ECM) targets thus they play crucial roles in tissue remodeling and tertiary dentine formation [1, 5, 6]. MMP-8, a collagenase, cuts native triple helix type 1 collagen fibrils into three fourth and one fourth fragments. From their localization in mineralized dentine, it is supposed that MMP-8 plays an important role in the organization of the dentine matrix prior to mineralization during tooth development [7]. MMPs also take part in homeostatic cytokine and cell surface proteoglycan regulation [1].

In addition to their function in physiology, MMP-2, MMP-8, and MMP-9 are believed to be involved in various dental pathologies too. In pulpitis, they are suspected to be involved in ECM degradation and growth factor release [8–10]. In caries progression, MMP-2, MMP-8, and MMP-9 have been shown to facilitate dentine matrix breakdown after demineralization [2, 3, 11]. Aside from in situ MMPs, pulp-derived MMPs have also been suggested to contribute through the dentinal fluid [3].

Resin-based composites (RBC) and their respective bonds are a mixture of resin monomers polymerized by an appropriate wavelength of light. RBC restorations and especially the application of adhesives have been associated with pulp inflammation and higher secondary caries rate, when compared with amalgam [12–14]. Current material designs rarely ensure a conversion degree exceeding 75%, resulting in the presence of leachable monomers such as triethylene-glycol dimethacrylate (TEGDMA), which can reach the pulp [15]. Increased gelatinolytic activity in “caries-affected” dentine tubules under composite restorations may facilitate TEGDMA reaching the pulp [16]. Efforts concerning TEGDMA toxicity in pulp tissue have focused on glutathione depletion, DNA damage and redox alteration [17–19], but the effect on MMPs, as possible contributors to both secondary caries and pulpal inflammation, is incompletely understood. The effect of different adhesives on pulp-derived and in situ dentine MMPs has been described [20–23]. Results suggest that such collagenases/gelatinases could contribute to the breakdown of the resin-dentine interface thereby causing the early failure of restorations. However, few studies exist attributing MMP activity and production changes, to specific leachable resin components. TEGDMA has been shown to inhibit the activity of MMP-2 extracted from mouse gingival tissue [24]. While, hydroxyethyl methacrylate (HEMA) inhibited MMP-2 and MMP-9 activity and production in pulp-derived cells [25]. Few gene expression studies have found MMP-1, MMP-3, MMP-10, and MMP-12 increases in pulp cells upon exposure to low toxic TEGDMA concentrations [26, 27]. However, there appears to be a lack of studies showing TEGDMA effect specifically on dentally relevant MMP-2, MMP-8, and MMP-9 in pulp cells. Moreover, elucidating concurrent signal transduction alterations would add valuable information to the current understanding of monomer toxicity and identify molecules with a possible role in MMP increase. TEGDMA effect on various signaling molecules, especially mitogen-activated protein kinase (MAPK) cascades has been reported [28, 29]. The activation of extracellular signal-regulated kinase (ERK) and p38 have been suggested to play a role in increasing MMP-2, MMP-8, and MMP-9 production in pulp cells stimulated by TNFα and certain chemokines [30, 31]. The induction of such molecules concurrently with TEGDMA-triggered MMP changes has not been demonstrated yet.

In light of the above, the aim of thi*s* in vitro investigation is to establish a possible effect of low TEGDMA monomer concentrations on MMP-2, MMP-8, and MMP-9 production and total collagenase/gelatinase activity in pulpal cells, by employing western blotting, immunohistochemistry, and a specific activity assay while also examining concurrently ERK1 and ERK2, p38, and c-Jun N-terminal kinase (JNK) activation to confirm the previously suggested role of these signaling molecules in the monomer-induced stress response. It is hypothesized that sublethal concentrations of TEGDMA increase MMP activity and/or production, which could be a further component in the mechanisms of intrapulpal monomer toxicity.

## Materials and methods

### Reagents

All chemicals used were obtained from Sigma-Aldrich (now Merck KGaA, Darmstadt, Germany) unless stated otherwise.

### Pulp cell culture

Pulp tissue was collected from five healthy third molar teeth extracted for orthodontic reasons. Informed consent was obtained under a protocol approved by the University of Pecs (Pecs, Hungary, under license No. PTE3026/2007). In line with patient confidentiality guidelines, all data were anonymized and study was performed in accordance with the ethical standards laid down in 1964 Declaration of Helsinki and its later amendments or comparable ethical standards.

Following extraction, pulp tissue was removed according to Sun et al. [25]. Through an explant method, cells were cultured in minimum essential medium Eagle-alpha modification (Alpha MEM) containing ultraglutamine 1, ribonucleosides, and deoxyribonucleosides (Lonza, Basel, Switzerland) with the addition of 10% fetal bovine serum (FBS, Euroclone, Milan, Italy) and antibiotics (100 U/ml penicillin, 100 μg/ml streptomycin, 2.5 μg/ml amphotericin B) and cultured in a humidified atmosphere containing 5% CO_2_ at 37 °C. As the cultures reached 90% confluence, a passage was performed to plastic Petri dishes. Cell cultures were first washed with phosphate-buffered saline (PBS, 1.37 mM NaCl, 0.27 mM KCl, 0.43 mM Na_**2**_HPO_4_·7H_**2**_O, 0.14 mM KH_**2**_PO_4_, pH 7.4) followed by a 10-min trypsin (0.25% trypsin + 0.02% ethylene-diamine-tetraacetic acid (EDTA); Gibco, Grand Island, NY, USA) digestion in a controlled, 37 °C, environment. After two or three passages, cells were seeded at an arbitrary density of 2 × 10^4^ cells/cm^2^, based on previous experience on similar populations. Forty-eight hours prior to the start of TEGDMA exposure, the medium was exchanged to 2% FBS containing medium (without antibiotics) in order to decrease the potential signaling interference.

### Monomer exposure

Cells were exposed to TEGDMA concentrations of 0.1, 0.2, and 0.75 mM for 24 h as suggested by our pilot viability studies. For preliminary viability testing, a 1-day exposure to concentrations of 0.1, 0.2, 0.75, 1.5, and 3 mM was carried out in order to find out if the concentration range has not yet cause significant cell death.

### WST-1 colorimetric viability assay

Water-soluble tetrazolium salts (WST-1) colorimetric assay was performed as an indicator of intracellular mitochondrial metabolism and hence viability. After TEGDMA exposure, the medium was removed and 200 μl of WST-1 reagent (Hoffmann-La Roche, Basel, Switzerland) was added in a 1:9 WST to 2% Alpha MEM medium ratio (180 μl of medium and 20 μl of WST dye). The cells were stored at 37 °C for 4 h followed by transfer to a 96-well plate. Absorbance was measured in 100 μl samples at 440 nm by a FluoStar Optima plate reader (BMG Labtech, USA).

### EnzCheck Gelatinolytic/Collagenolytic activity assay

EnzCheck Gelatinolytic/Collagenolytic activity assay kit (Molecular Probes, Eugene, OR, USA) was used to investigate the possible effect of various concentrations of TEGDMA monomers on enzyme activity in pulp cell lysates and media derived from cells. Following the manufacturer’s instructions, the highly labeled fluorescent gelatin substrate was mixed with reaction buffer in a final volume of 200 μl. Cell culture media or dilutions of mechanically homogenized cell extracts suspended in lysis buffer were collected into 96-well plates. Proteolysis was determined by a Promega Glo Max plate reader (Madison, Wisconsin, USA), operated at a fluorescent excitation maximum of 495 nm and emission maximum of 515 nm. Absolute enzyme activity values were plotted and analyzed.

### Western blotting

After TEGDMA treatment, cells were harvested and lysed as described in previous studies [32]. Briefly, the pulp cells were collected in cold lysis buffer (50 mM Tris-base, pH 7.4, 10% glycerol, 150 mM NaCl, 1 mM EGTA, 1 mM Na-orthovanadate, 100 mM NaF, 5 μM ZnCl_2_, 10 μg/ml aprotinin, 1 μg/ml leupeptin, 1 mM PMSF, 1% Triton X-100) and homogenized for 20 s. The homogenate was centrifuged at 4 °C and at 40,000×*g* for 30 min. Protein concentrations of the supernatants were measured (Lowry’s method, Detergent Compatible Protein Assay Kit, Bio-Rad, Hercules, CA, USA). Protein concentrates were then diluted to contain equal 30 μg of protein. Laemmli buffer (prepared from 25 ml 1 M Tris-HCl, pH 6.8, 40 ml glycerol, 8 g SDS, 10 ml 100 mM EGTA, 10 ml 100 mM EDTA, 1 ml 1% bromophenol blue, and distilled water to a total volume of 100 ml) was added followed by boiling for denaturation. Subsequently, 10% SDS-containing polyacrylamide gels were used to separate proteins based on molecular size. Proteins were then transferred to PVDF membranes (Hybond-P, GE Healthcare, UK) by the Trans-Blot Turbo system (Bio-Rad, Hercules, CA, USA). Nonspecific binding on the membrane was blocked by 3% nonfat dry milk in TBS-Tween (10 mM Tris-base, 150 mM NaCl, 0.2% Tween-20, pH 8.0). Rabbit polyclonal primary antibodies were added specific to MMP-8 (Thermo Fisher Scientific, Waltham, MA, USA); MMP-9 (Abcam, Cambridge, UK); p-(phospho-)ERK1/2 (Thr202/Tyr204), p-p38 (Thr180/Tyr182), and p-JNK (Thr183/Tyr185) (Cell Signaling Technology, Beverly, MA, USA); and rabbit monoclonal antibodies specific to MMP-2 (D4M2N, Cell Signaling Technology, Beverly, MA, USA) diluted 1:1000 in the blocking solution and then incubated overnight. Excess antibody was removed by five washes of TBS-Tween. Incubation with a horseradish-peroxidase (HRP)-conjugated polyclonal goat anti-rabbit secondary antibody (Pierce, Thermo Fischer Scientific, Rockford, IL, USA) diluted 1:10,000 in blocking solution followed. Enhanced chemiluminescent signal (Immobilon Western, Millipore Corporation, Billerica, MA, USA) was detected using a G:box gel documentation system (Syngene International Ltd, Bangalore, India). Membranes were then chemically stripped of antibodies (0.2 M glycin-HCl, 0.2% Tween-20, 0.05%, pH 2.5) and reprobed using β-actin, ERK1/2, p38, or JNK (Cell Signaling Technology, Beverly, MA, USA) rabbit polyclonal primary antisera as mentioned above to prove the absence of disparity in protein concentration among samples. Densitometry analysis was performed using the ImageJ software (National Institutes of Health, USA).

### Immunofluorescence microscopy

Pulp cells seated onto glass coverslips were exposed to the conditioned or control media and processed for immunocytochemistry. Briefly, a quick rinse in 37 °C PBS was followed by a 4% paraformaldehyde fixation in PBS at pH 7.4, 4 °C for 12 h. The fixative was removed by three changes of TBS (50 mM Tris-HCl, pH 7.4, 150 mM NaCl). Permeabilization was achieved by a 30-min wash with 0.1% Triton X-100 in TBS at 4 °C. Nonspecific binding sites were blocked by incubation in 5% nonfat dry milk in TBS at 4 °C for 1 h. The primary antibodies MMP-2 (D4M2N, Cell Signaling Technology, Beverly, MA, USA), MMP-8 (Thermo Fisher Scientific, Waltham, MA, USA), and MMP-9 (Abcam, Cambridge, UK) were diluted 1:100 in the blocking solution and incubated with the cells overnight at 4 °C. After five washes in TBS, Cy3-conjugated polyclonal donkey-anti-rabbit antibodies (Jackson Immuno Research, Cambridgeshire, UK) were diluted 1:200 in the blocking solution and added to the cells for overnight incubation at 4 °C. For each antigen respectively, control samples prepared by the omission of the primary antibodies produced no visible fluorescence signal using the same microscope. During the final five TBS washes, nuclei were counterstained with Hoechst 33342 (Calbiochem, La Jolla, CA, USA). Fluorescence microscopy images were obtained using an Olympus FV-1000 laser scanning confocal system (Olympus Europa, Hamburg, Germany). The presented single optical slice pictures were generated using a × 40 dry objective and are representative of series of four to five independent experiments with similar results. Determination of raw integrated density values was carried out using the ImageJ software (National Institutes of Health, USA).

### Plotting of experimental data and statistical analysis

WST-1, collagenolytic activity assay, western blot, and immunocytochemistry density data presented in diagrams were gathered in a series of four independent experiments; values shown are the means and standard deviations (± SD). Significance of differences was determined using one-way analysis of variance (ANOVA) test with the application of Tukey post hoc tests for multiple samples. *P* values ˂ 0.05 were considered to be significant. Significant differences considered relevant to major findings were marked in the graphs, and their corresponding *P* values were indicated in the figure legend.

## Results

### WST-1 colorimetric viability assay

**Fig. 1:**
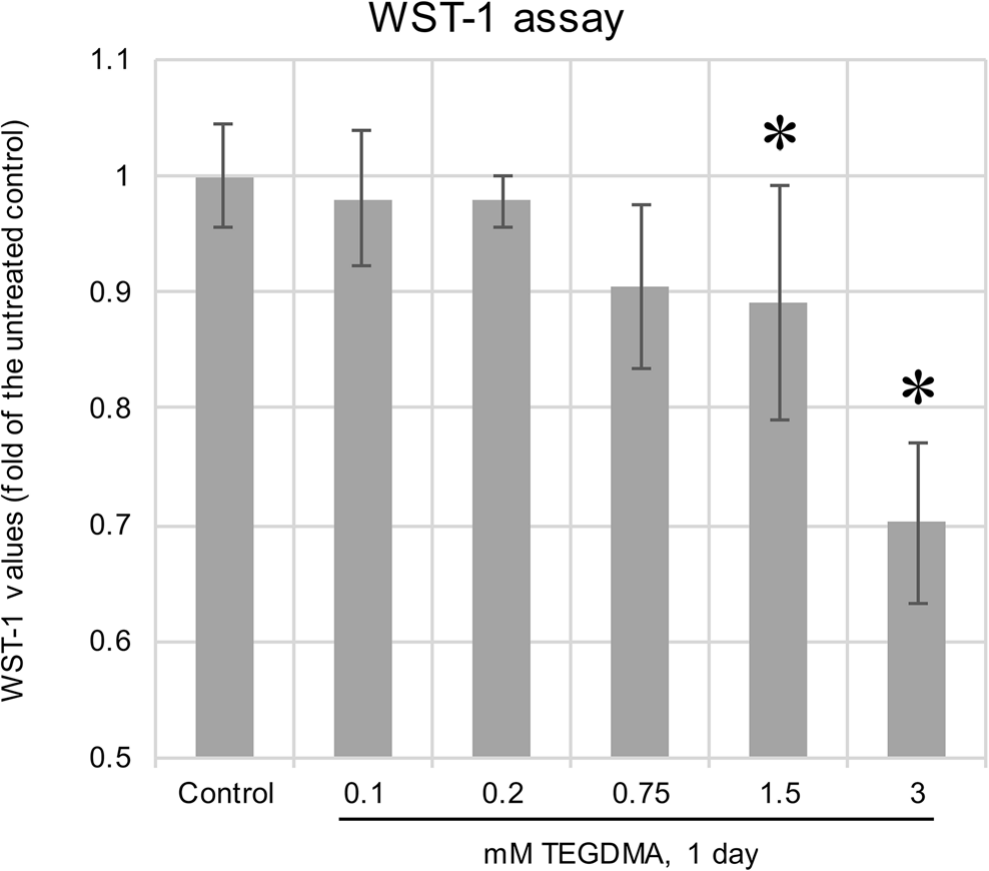
Viability changes in pulp cells after a 1-day exposure to 0.1, 0.2, 0.75, 1.5, and 3 mM TEGDMA as detected by a water-soluble tetrazolium-1 assay. The graph represents the WST-1 values as a ratio compared with the average value of the day 1 control. *Significantly different from the untreated control of day 1 (*P* = 0.0251 and *P* < 0.0001 at 0.75 and 3 mM TEGDMA concentrations, respectively)

The time- and dose-dependent effect of TEGDMA on cell viability was investigated by a WST-1 assay (Fig. 1). Results show, after 24 h, a significant decrease in viability upon exposure to 1.5 and 3 mM TEGDMA, while 0.1, 0.2, and 0.75 mM did not affect viability in a significant manner; 2nd- and 5th-day results proved to be erratic, and extermination of cells was apparent at 3 mM by the 2nd day and at 1.5 mM by the 5th day (data not presented). Therefore, the highest concentration and longest treatment time applicable for further experiments was decided to be 0.75 mM and 1 day, which are conditions not yet causing substantial cell death.

### Gelatinolytic/Collagenolytic activity assay

**Fig. 2:**
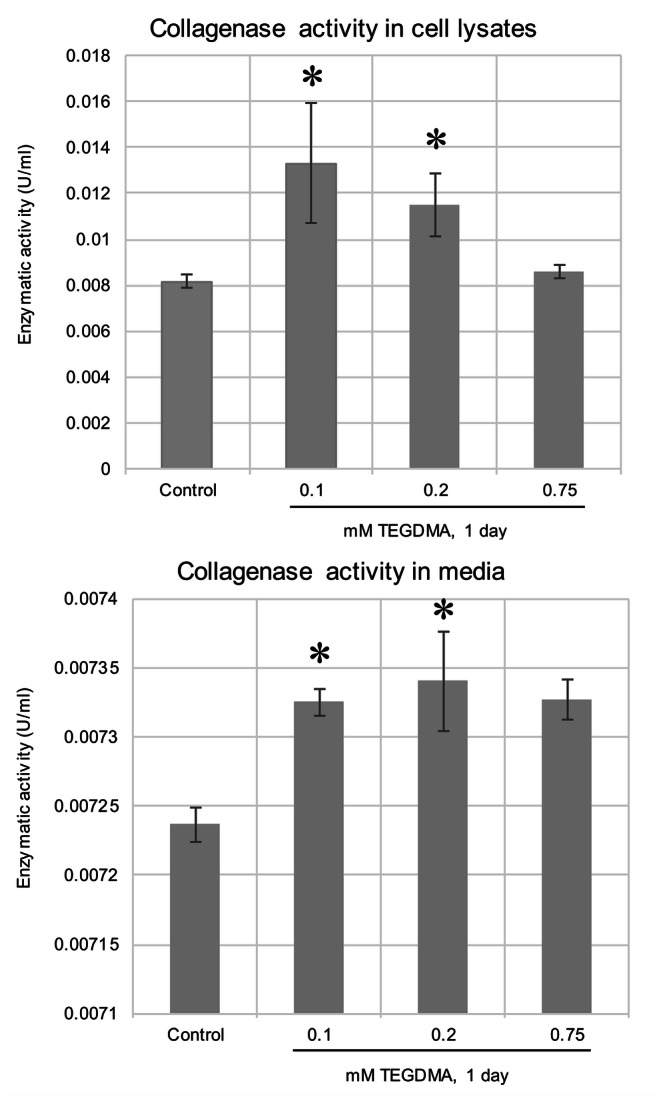
Total collagenase/gelatinase activity after exposure to 0.1, 0.2, and 0.75 mM TEGDMA. The diagram represents enzyme activity values detected by a specific kit from lysates of pulp cells and the media removed from cultures. *Significantly different from the day 1 untreated control in cell lysate sample (P = 0.002 and P = 0.0381 for 0.1 and 0.2 mM, respectively), from 1st day untreated control in medium sample (P = 0.0003, P < 0.0001, and P = 0.0003 for 0.1, 0.2, and 0.75 mM, respectively)

Compared with the control, pulp cell lysates showed after 1 day, a mild increase in total collagenase/gelatinase activity upon exposure to 0.1 and 0.2 mM TEGDMA (Fig. 2) (also from media upon 0.75 mM TEGDMA exposure). Longer exposures resulted in a stagnation and decrease in collagenase/gelatinase activity both from the lysates and medium for all groups including the control (results not presented).

### Western blotting

**Fig. 3:**
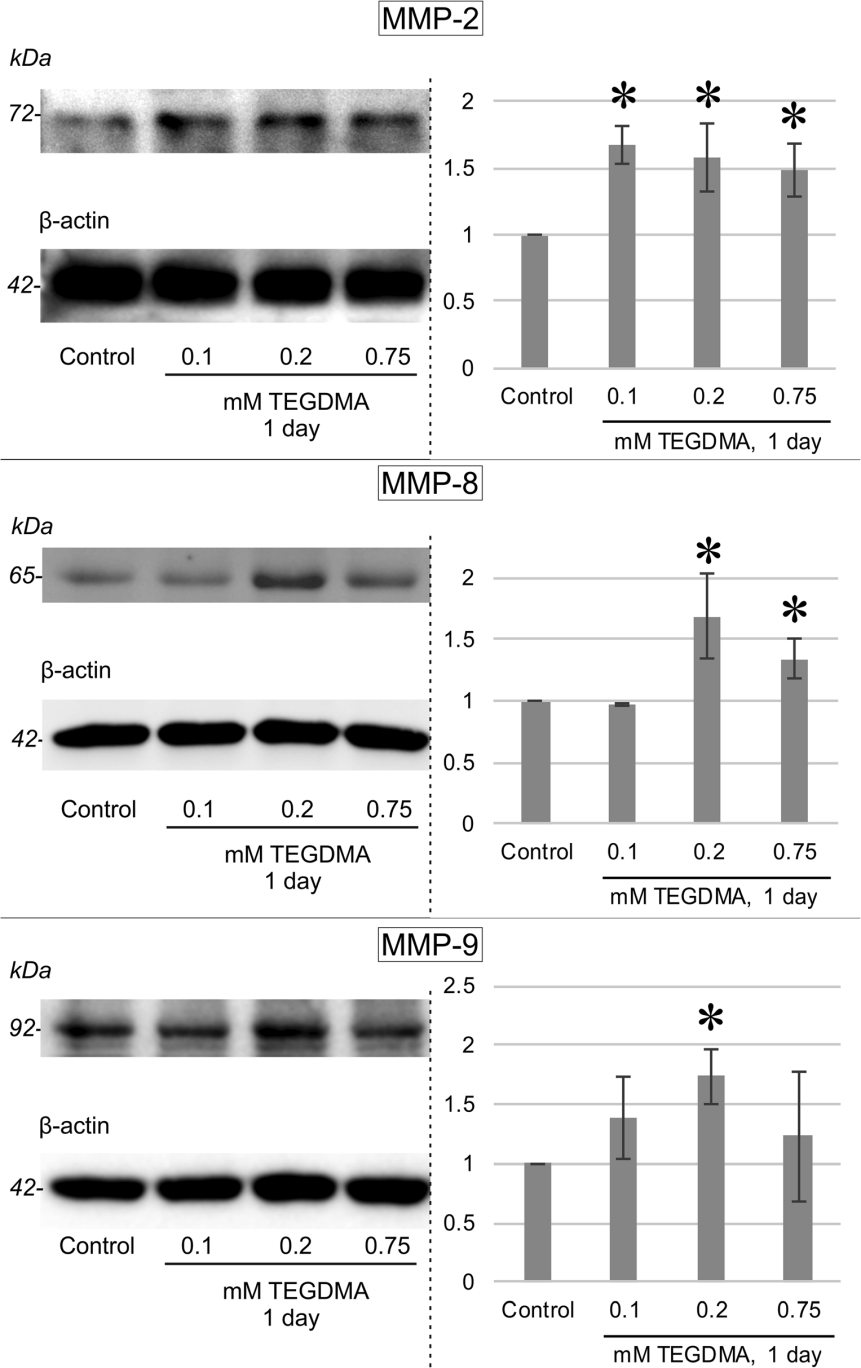
Immunoblots illustrating the changes in MMP-2, MMP-8, and MMP-9 concentrations in pulp cells upon a 1-day exposure to TEGDMA concentrations of 0.1, 0.2, and 0, 75 mM. β-Actin bands are results of western blot membrane reprobing which served as loading control each time. Results of the quantitative analysis of densitometry data gathered by ImageJ are presented on the right side. *Significantly different from the 1-day untreated control; in the case of MMP-2, *P* = 0.0026, 0.0079, and 0.0249 at 0.1, 0.2, and 0.75 mM, respectively; for MMP-8, *P* < 0.0001 and *P* = 0.0410 at 0.2 and 0.75 mM, respectively; and for MMP-9, *P* = 0.0021 at 0.2 mM

TEGDMA exposure also led to increased levels of MMP-2, MMP-8, and MMP-9 in pulp cells, as detected by the western blot analysis (Fig. 3). The lowest TEGDMA concentration of 0.1 mM caused an increase in MMP-2 production only. A strong increase in all tested MMPs was seen after exposure to 0.2 mM TEGDMA; 0.75 mM TEGDMA increased MMP-2 and MMP-8 levels marginally, without influencing MMP-9 production. The presented photos are representative of series of four to five independent experiments with similar results.

### Immunofluorescence microscopy

**Fig. 4:**
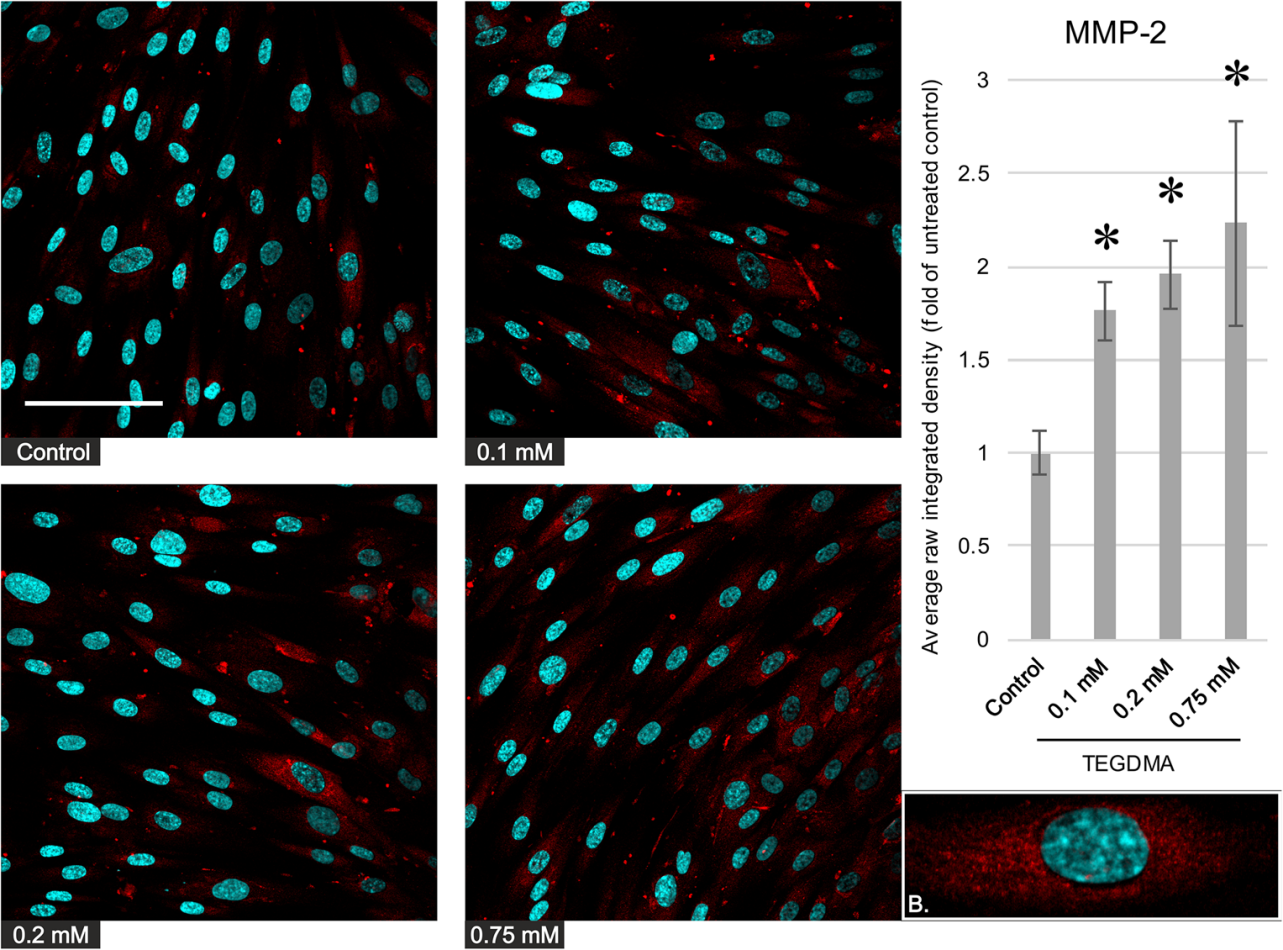
Left, Immunocytochemistry images depicting the level and distribution of MMP-2 (red) in pulp cells after a 1-day exposure to 0.1, 0.2, and 0.75 mM TEGDMA; nuclei were counterstained with Hoechst 33342 (blue). The scalebar represents the length of 100 μm. Right, average fluorescence intensity values of images gathered by analysis using the ImageJ software, compared with the untreated control. *Significantly different from the 1-day untreated control, *P* = 0.0191, 0.0039, and 0.0005 at 0.1, 0.2, and 0.75 mM, respectively. B, a representative cutout magnified image showing the intracellular distribution of the antigen (from the control picture)

**Fig. 5:**
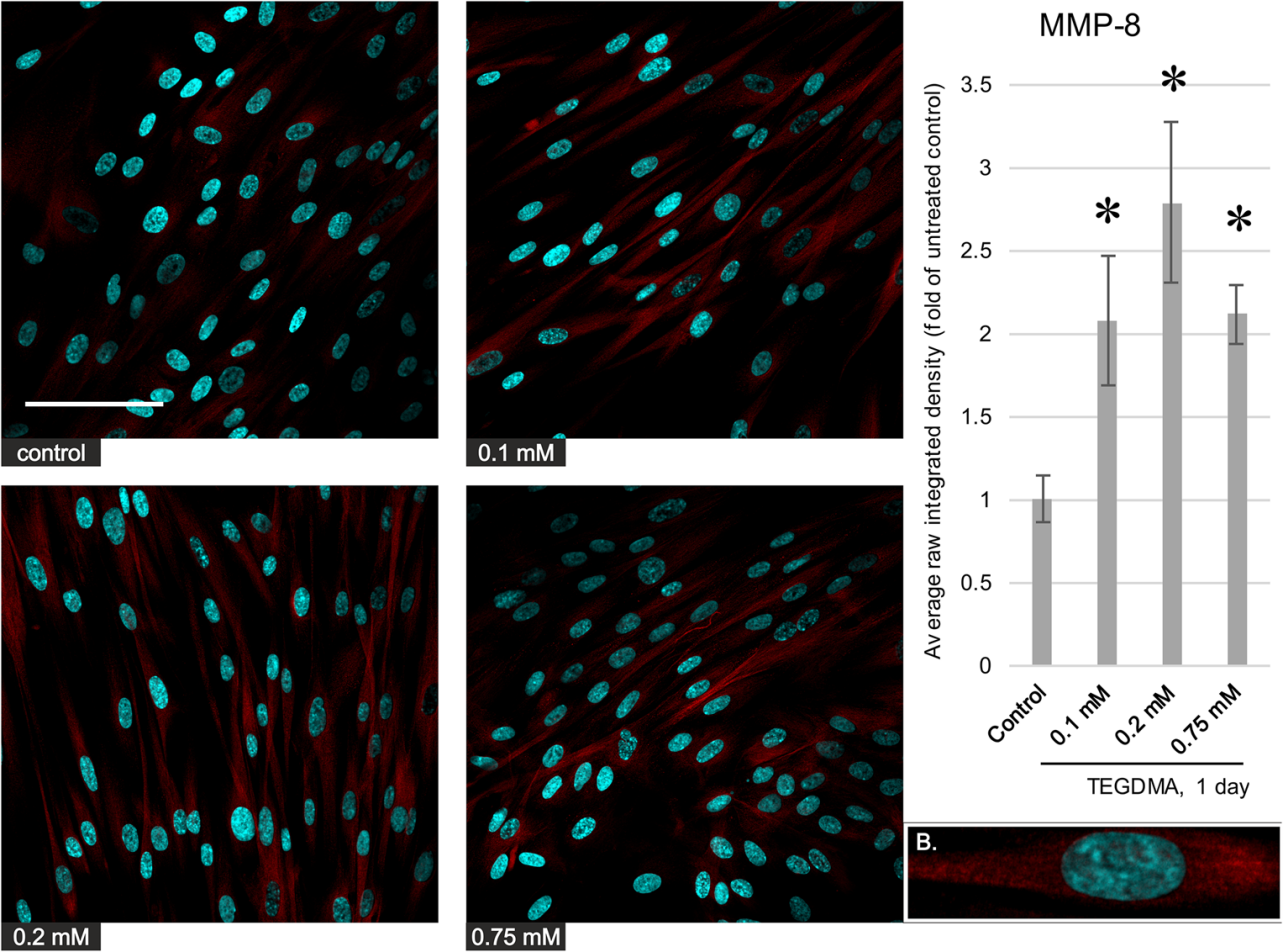
Left, immunocytochemistry images depicting the level and distribution of MMP-8 (red) in pulp cells after a 1-day exposure to 0.1, 0.2, and 0.75 mM TEGDMA; nuclei were counterstained with Hoechst 33342 (blue). The scalebar represents the length of 100 μm. Right, average fluorescence intensity values of images gathered by analysis using the ImageJ software, compared with the untreated control. *Significantly different from the 1-day untreated control, *P* = 0.0028, 0.0001, and 0.0021 at 0.1, 0.2, and 0.75 mM, respectively. B, a representative cutout magnified image showing the intracellular distribution of the antigen (from the 0.2-mM picture)

**Fig. 6:**
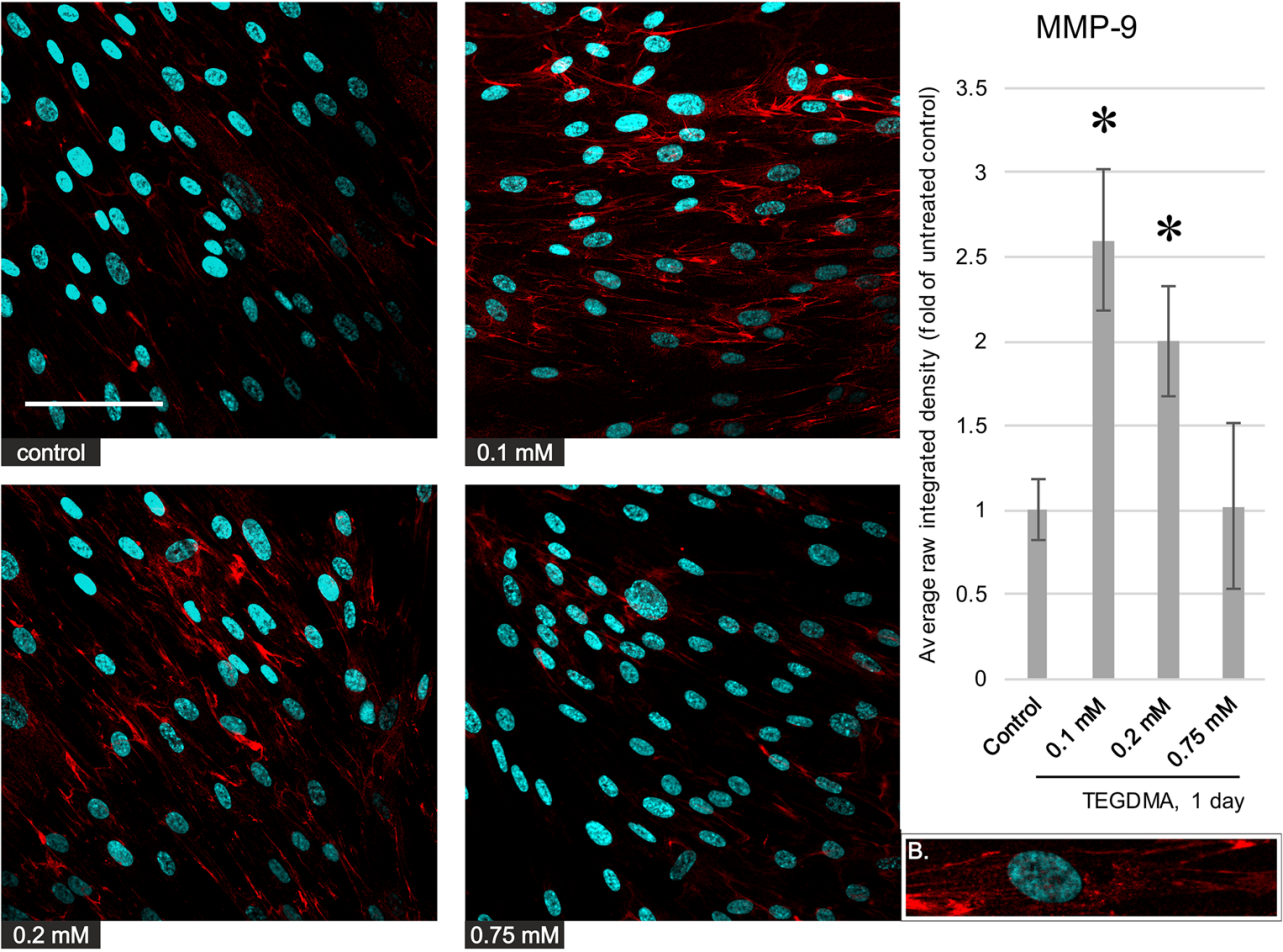
Left, immunocytochemistry images depicting the level and distribution of MMP-9 (red) in pulp cells after a 1-day exposure to 0.1, 0.2, and 0.75 mM TEGDMA; nuclei were counterstained with Hoechst 33342 (blue). The scalebar represents the length of 100 μm. Right, average fluorescence intensity values of images gathered by analysis using the ImageJ software, compared with the untreated control. *Significantly different from the 1-day untreated control, *P* = 0.0003 and 0.0125 at 0.1 and 0.2 mM, respectively. B, a representative cutout magnified image showing the intracellular distribution of the antigen (from the 0.1-mM picture)

Immunocytochemistry was also performed to further confirm the findings acquired by western blot and to investigate possible changes in antigen distribution. While untreated cells labeled weakly or not at all for the MMPs, exposure to 0.1, 0.2, and 0.75 mM TEGDMA resulted in increased MMP-2, MMP-8, and MMP-9 immunostaining in pulp cells (Figs. 4, 5, and 6) with the exception of the 0.75-mM treatment and MMP-2 levels. In the case of MMP-2 and MMP-8, the antigens showed a cytoplasmic distribution, with a grainier appearance for MMP-8. MMP-9 produced a grainy, cytoplasmic as well as a filamentous signal, the latter often in the vicinity of the cell membrane. There was no detectable difference in antigen localization between the control and treated pulp cells.

### ERK, p38, and JNK activation

**Fig. 7:**
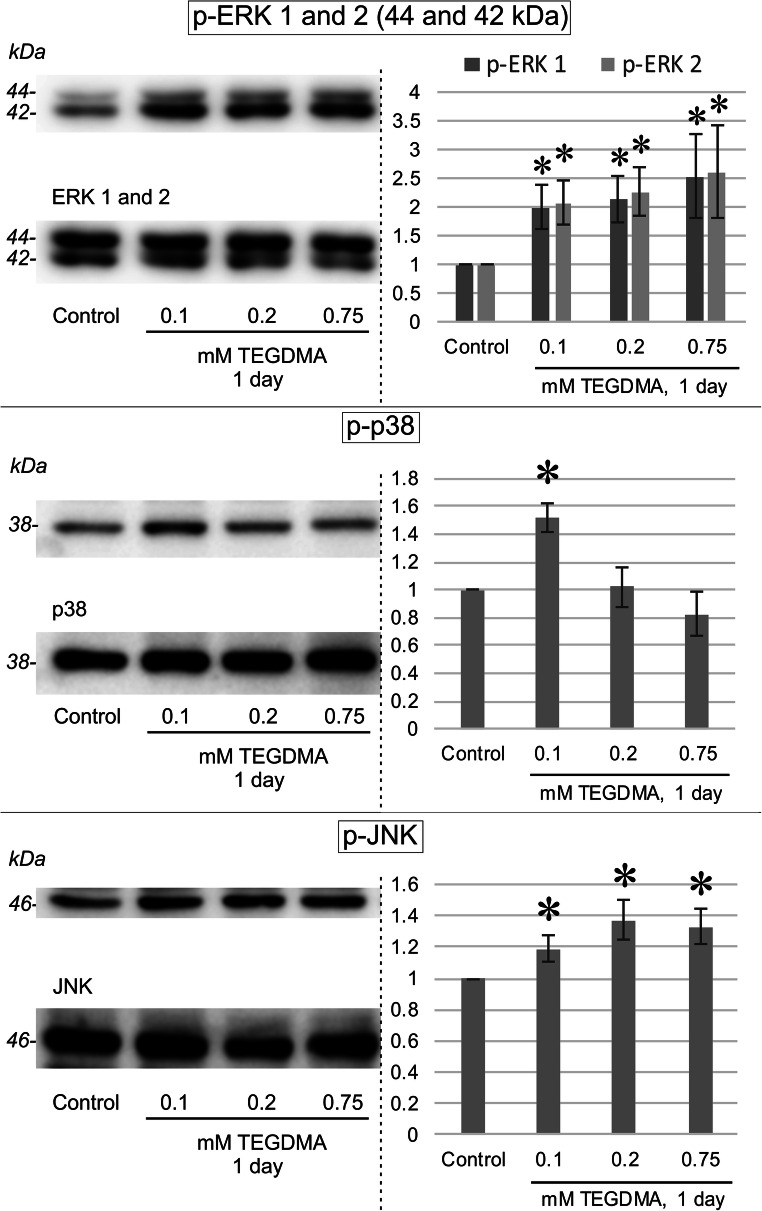
Immunoblot of changes in the concentrations of 42/44 kDa versions of phosphorylated (activated) ERK, p38, and JNK in pulp cells after a 1-day exposure to 0.1, 0.2, and 0.75 mM TEGDMA. The membranes were reprobed using anti-ERK1/2, anti-p38, and anti-JNK antibodies for the purpose of loading controls. The equal level of proteins throughout control and treated pulp cells in cases of JNK, p38, ERK, respectively, shows that the increased p-ERK, p-JNK, and p-p38 concentrations upon TEGDMA treatment is the result of the activation (phosphorylation) of these signaling proteins and not a change in their amount. Results of the quantitative analysis of densitometry data gathered by ImageJ are presented on the right side. *Significantly different from the 1-day untreated control; in the case of p-ERK 1, P = 0.0345, 0.0149, and 0.0013 at 0.1, 0.2, and 0.75 mM, respectively; for p-ERK 2, P = 0.0131, 0.033, and 0.0003 at 0.1, 0.2, and 0.75 mM, respectively; for p-p38, P < 0.0001 at 0.1 mM; for p-JNK, P = 0.025, P < 0.0001, and P = 0.0001 at 0.1, 0.2, and 0.75 mM, respectively

The identification of concurrent signaling pathway activation was also attempted. Western blotting (Fig. 7) showed after 1 day increased levels of both 44 and 42 kDa variants of phosphorylated (activated) ERK as well as p-JNK, for all tested TEGDMA concentrations. A considerable p38 activation could only be seen after exposure to 0.1 mM TEGDMA. After the removal of antibodies from the blots, ERK1/2, p38, and JNK proteins were detected on the corresponding membranes using the proper antisera.

## Discussion

Polymerization degree of resin-based composite restorative materials correlates well with the quantity of leached monomers [33, 34]. Leaching typically happens in the first 24 h but continues up to a month. Monomers have been shown to reach the pulp, with 4 mM being quoted as a worst-case intrapulpal concentration [15]. Clinical observations suggest that monomer-containing adhesives/RBCs may lead to pulp inflammation [12, 13] and MMP induction [23]. Pulp-derived MMPs have been suggested to play a role in caries [2], hybrid layer degradation [14], as well as pulpal inflammation [10]. With a lack of studies examining the effect of specific adhesive/RBC components on MMP activity, the aim of this study was to explore how TEGDMA might affect MMP expression, collagenase/gelatinase activity, and what signaling molecules may play a role.

In this experimental design, pulp cells were exposed to 0.1, 0.2, and 0.75 mM TEGDMA solution for 24 h. Exposure time and concentrations were chosen due to the substantial cell death which was seen for all treated populations after 48 h and above concentrations of 0.75 mM in our pilot studies. Regarding the employed concentration and study time-frame, similar exposure conditions were used also in other studies to detect gene expression changes and production of specific proteins, all of which yielded representative findings [28, 35].

Results of this investigation suggest that cells exposed to sublethal TEGDMA concentrations for 24 h show a mild increase in total collagenase/gelatinase activity, as well as a rise in MMP-2, MMP-8, and MMP-9 production, thereby prompting to accept the authors’ hypothesis. Few studies, if any, so far have explicitly aimed at investigating possible intrapulpal MMP quantity changes upon TEGDMA exposure. Some, however, have documented changes in these proteins as findings in gene expression studies. Torun et al., in their study on pulp cells, showed that 1 mM TEGDMA caused a 10-fold increase in MMP-10 and MMP-12 expression, as measured by DNA microarray and real-time PCR [26]. Similarly, although only a fraction of RNA transcripts translates to proteins, Cho et al. detected elevated levels of MMP-1 and MMP-3 RNA in human dental pulp cells after a 48-h exposure to low-toxic, 1.3-mM, TEGDMA [27]. The current study has identified MMP-2, MMP-8, and MMP-9 production to increase in a nonlinear way with applied TEGDMA concentrations. Currently, the mechanisms for TEGDMA-caused MMP production can only be speculated. A possible explanation could be signaling alterations taking place in the background. TEGDMA seems to influence intracellular signal transduction through direct alterations in MAPK cascades [28, 29]. The present study also demonstrated an increased activation of MAPK members ERK1/2, JNK, and p38 with the concurrent increase in MMPs. p38 and JNK are activated by oxidative stress and proinflammatory cytokines, while ERK—downstream of Raf—mostly mediates growth factor effects leading to cell differentiation, survival, and migration [36]. No literature has been found confirming the function of the abovementioned signaling molecules in TEGDMA-driven MMP production. However, data exists showing the direct involvement of ERK1/2 in inducing MMP-2 expression in deciduous pulp cells, triggered by TNFα [37]. The concurrent activation of signaling molecules and induction of MMP expression found in the present experimental set-up cannot confirm the role of p38, JNK, or ERK1/2 in MMP increase. Considering the abovementioned roles of these signaling molecules, they may only be part of a broader stress response.

These findings are in line with those of Lehmann et al. who observed an elevation in MMP-2 and pro-MMP-9 expression in the pulp upon self-etching adhesive treatment of tooth slices and speculated this to be caused by either the acidity or the monomer content [22]. In a later study, an increase in MMP-2 expression was also seen after direct treatment of human pulp cells with various adhesives. Comparatively, due to the lack of acidity in the latter study, this author attributed the observed effect to the monomer content [23]. Interestingly, the current findings are in contradiction with those of Sun et al. who found HEMA, another leachable monomer, to inhibit MMP-2 and MMP-9 expression in pulp cells, thereby suggesting a potential hybrid-layer protective role to this constituent [25].

The second finding of this study is the increase in total collagenase/gelatinase activity—mostly due to matrix metalloproteinases [38]—seen in pulp cells upon TEGDMA treatment. The employed EnzCheck assay uses clostridium histolyticum collagenase as reference. Bacterial cleavage sites could be different from human enzymes’; however, prior studies have shown it to be a useful model in dental in vitro inhibition investigations [39, 40]. Activity measurements at later time points were disregarded due to the concurrent drop in viability detected in all groups by the supplementary WST-1 stain*.* Few theories exists addressing MMP activation. MMP activity in the propeptide form is limited by a cysteine sulfhydryl group which interacts with a zinc ion in the catalytic domain of the enzyme [1]. Scission by another protease or removal of this interaction, by a conformational “cysteine” switch, is a key step in enzyme induction. MMPs have been shown to be activated in acidic pH [40], which led to the speculation that the mildly acidic monomers may be responsible for adhesive-induced MMP activity increase [21, 23]. Tissue inhibitors of metalloproteinases (TIMPs) are also believed to physically regulate MMPs by occupying active sites [41]. From the correlation between cathepsin levels and MMP activity, this protein has also been suspected to be involved in MMP activity regulation [42]. The mechanism for activity induction can only be speculated at this stage, but the concurrent detection of JNK, p38, and ERK activation with the rise of total collagenase/gelatinase activity in the current study may suggest a role for signaling in enzyme activation. Further studies would be required to elicit possible TEGDMA effect on the previously mentioned proteins in pulp cells.

Although TEGDMA induction of collagenase/gelatinase activity has hardly been demonstrated so far, the findings of the present study can be considered to agree with studies that found MMP activity to increase upon exposure to various adhesives [20, 21]. Interestingly, on the contrary however, there is also evidence suggesting that such monomers could inhibit MMP-2 and MMP-9 activity, by complex formation between the ether bonds of TEGDMA and Zn at the catalytic domain of MMPs [24, 43].

Regarding the limitations of the present study, demonstration of MMP changes with the application of ERK, p38, and JNK inhibitors would have provided additional data. A lack of MMP elevation in the presence of ERK, p38, and JNK inhibitors would have provided specific evidence for the involvement of these signaling molecules in triggering MMP increase. Therefore, based on the current experimental set-up, the role of these signaling molecules could only be suggestive and requires further investigation. Moreover, combinatorial studies involving two or more resin monomers, as present in commercially available RBCs, in combination and individually would have provided information not just about another constituent but also any potential synergistic effect which would apply better to the in vivo situation.

In the present study, TEGDMA was shown to increase the activity and expression of MMP-2, MMP-8, and MMP-9 in pulp cells in vitro with a concurrent increase in ERK, p38, and JNK activation, the specific roles of which are yet unclear. It can be speculated, based on current literature, that this MMP increase could play a role in caries, hybrid layer degradation, and in the alteration of intrapulpal dynamics of reparative dentin production and ECM degradation. Further in vivo studies should be done to confirm such effects and to quantify TEGDMA leakage to the pulp, since many factors such as high intrapulpal pressure and outward dentinal fluid flow, as well as chemical interactions with dentine could limit pulpal exposure in vital teeth.

## Conclusion

Within the limitations, in conclusion the current study has demonstrated that:

- Low concentrations of TEGDMA monomers (0.1 and 0.2 mM) cause a mild elevation in total collagenase/gelatinase activity, suggestive of MMP activation, in pulp cells.
- Monomer presence induces dentally relevant MMP-2, MMP-8, and MMP-9 production.
  Exposure to low concentrations of TEGDMA also led to the activation of ERK1/2, p38, and JNK. Specific roles of these signaling molecules in the stress response and/or MMP induction are yet to be determined.
